# Influence of Acute Phase Proteins on Neutrophil Function In Vitro

**DOI:** 10.1096/fba.2025-00148

**Published:** 2025-10-19

**Authors:** Richard F. Kraus, Isabell Wild, Michael A. Gruber, Martin G. Kees

**Affiliations:** ^1^ Department of Anesthesiology University Hospital Regensburg Regensburg Germany

**Keywords:** acute phase proteins, chemotaxis, NETosis, neutrophil function, ROS

## Abstract

As part of a systemic inflammatory response, acute phase proteins (APPs) are released into the blood to support the body's immune response. Once in the bloodstream, the APPs can also interact with immune cells, such as neutrophil granulocytes (PMNs). However, this interaction is not yet fully understood. This study aims to investigate the effects of specific APPs on various functions of neutrophil granulocytes in vitro. PMNs were isolated from peripheral blood of healthy volunteers and subsequently exposed to varying concentrations of CRP, fibrinogen, or ferritin. As activating agents, TNF‐α (TNFα)/*N*‐formylmethionine‐leucyl‐phenylalanine (fMLP), phorbol myristate acetate (PMA), or ionomycin were used. Triggered oxidative burst and the expression of surface antigens CD11b, CD62L, and CD66b were measured by flow cytometry. Live cell imaging (LCI) determined the influence of ferritin on migration behavior, time‐resolved MPO release, and NET formation. CRP had a certain, non‐significant activating effect on PMN oxidative burst and surface epitope expression. Ferritin led to a moderate increase in the oxidative burst, especially after activation with TNF‐α/fMLP, PMA, or ionomycin. Ferritin reduced PMN migration without TNFα and enhanced PMN migration in the presence of TNFα. Without TNFα, ferritin prolonged NETosis and had a certain dose‐specific effect on MPO release. Fibrinogen mainly influenced the expression of CD11b, CD62L, and CD66b. The observed effects of acute phase proteins on PMNs showed plausible, concentration‐dependent, and differential effects for the tested APPs, but only of moderate magnitude. Future experiments should focus on intracellular signaling pathways and on the determination of PMN gene expression profiles. Given the broad context in which APPs are elevated, their interaction with PMNs is of considerable scientific interest for a multitude of clinical conditions.

## Graphical abstract

Acute phase proteins (APPs) modulate neutrophil granulocyte (PMN) functions in a concentration‐dependent manner. While CRP showed minimal activation, ferritin altered the oxidative burst, and fibrinogen mainly affected surface marker expression. These moderate but distinct effects highlight the relevance of APP–PMN interactions in inflammation and warrant further investigation of underlying signaling pathways and gene expression changes.

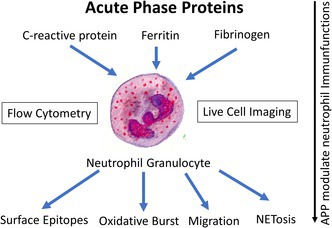

**Abbreviations**
APP: acute phase protein
CRP: c‐reactive protein
fMLP: *N*‐formylmethionine‐leucyl‐phenylalanine
MFI: median fluorescence intensity
MPO: myeloperoxidase
NET: neutrophil extracellular trap
PMN: polymorphonuclear cell
ROS: reactive oxygen species

## 1 Introduction

In case of an infection with systemic inflammatory response, cytokines such as interleukin‐1 (IL‐1), interleukin‐6 (IL‐6), and tumor necrosis factor (TNF) are released by stimulated macrophages and monocytes. As part of the acute phase response (APR), the body upregulates the basal temperature, and the liver increases the synthesis of various acute phase proteins (APP). The putative goal of the APR is to activate and support the body's defense functions in a very general sense, including e.g., coagulation and iron scavenging mechanisms. Moreover, the APR leads to leukocytosis, with neutrophil granulocytes (polymorphonuclear leukocytes, PMNs) making up the largest proportion in the peripheral blood [1]. Both PMNs and acute phase proteins are part of the innate immune system [2, 3]. In this study, the potential effects of APPs (C‐reactive protein (CRP), fibrinogen, and ferritin) on selected parameters of various neutrophil functions are to be examined in vitro. The structure, tasks, and known interactions of selected APPs investigated in this study with PMNs are described more in detail in the following paragraphs.

### 1.1 C‐Reactive Protein

C‐reactive protein was first detected in 1930 in the sera of patients suffering from acute pneumococcal infection [4]. It is present in the human body both as native, pentameric CRP (nCRP, with 5 subunits) and as modified, monomeric CRP (mCRP). However, the exact differences of both CRP isoforms with regard to their function during an inflammation are not well understood yet. CRP binds Ca^2+^‐dependently to ligands such as, for example, phosphocholine (PC), polysaccharide, and chromatin [5]. After binding to a suitable ligand, a rotation of the CRP's subunits occurs. This subunit rotation facilitates the interaction of various immune cells with CRP via the immunoglobulin receptors FcγRI and FcγRII [5, 6]. Thus, it also binds to the Fc receptors of PMNs, whereby the affinity to FcγRII appears to be higher than to FcγRI [7]. CRP has the ability to promote agglutination, complement binding, bacterial lysis, and phagocytosis [8]. It is regulated by cytokine IL‐6 [9] and in physiological conditions, the plasma level of CRP is less than 1 mg/L [5]. In case of bacterial or viral infections, the CRP concentration in the blood can increase up to > 100‐fold within 48 h [10].

### 1.2 Ferritin

Ferritin is an iron storage protein with an outside diameter of approximately 12 nm, which is typically composed of 24 subunits (polypeptide chains). There are two different subunit types, the so‐called high (heavy, H) and low (light, L) chains. Ferritin can consist of different ratios of subunit types. Thus, there are different isoforms (H24L0, H22L2, etc.) that are distributed in a tissue‐specific manner. The subunits jointly form a protein cage structure, i.e., a spherical protein shell [9, 11]. Ferritin is produced in various organs, such as the liver and kidneys, but also by macrophages. In the process, the macrophages recycle heme from erythrocytes [11, 12]. Thus, after stimulation with IL‐1 and tumor necrosis factor alpha (TNF‐α), the macrophages produce more ferritin as part of an acute phase response. In addition, these cytokines directly lead to an increased lysis of erythrocytes, which results in the release of more heme. The heme is then increasingly  phagocytosed by macrophages. Thus, Fe^2+^ ions are liberated, which have to be incorporated into ferritin [12, 13]. In healthy people, the ferritin concentration in the blood is between 20 and 300 ng/mL. In various diseases, the concentration can rise to over 1000 ng/mL or even exceed 10,000 ng/mL (in various autoimmune diseases) [12, 14]. A low ferritin value is an indicator of iron deficiency, e.g., in cases of chronic blood loss [1]. The studies on the ferritin‐PMN interaction are mostly from the old millennium and are quite unspecific. There are reports that describe that ferritin‐associated iron induces neutrophil dysfunction in hemosiderosis [15] or PMA‐stimulated PMNs can cause lipid peroxidation of erythrocyte membranes in the presence of ferritin [16]. Moreover, the interaction between superoxide produced by PMNs and ferritin seems to play a role in the pathogenesis of rheumatoid arthritis (RA) by releasing iron from ferritin, which can then contribute to the formation of harmful hydroxyl radicals [17].

### 1.3 Fibrinogen

Fibrinogen is a glycoprotein consisting of two groups of A alpha, B beta, and gamma chains [18]. It is a protein of the coagulation cascade and thus part of hemostasis. During hemostasis, fibrinogen is proteolytically cleaved by thrombin to fibrin, which ultimately forms a fibrin scaffold and thus part of the base for a thrombus. Fibrinogen has many binding sites, some of which are available for interactions already in uncleaved fibrinogen. However, some binding sites are only exposed after the cleavage to fibrin. Via these interaction sites, fibrin(ogen) can interact with other hemostatic factors such as thrombin or factor XIII, with pro‐ and antifibrinolytic proteins, extracellular matrix proteins, and cellular receptors [19]. Fibrinogen can bind to the leukocyte integrins MAC‐1 and α_x_β_2_ (CD11c/CD18) that are expressed on neutrophils, monocytes, and macrophages [20]. The binding via MAC‐1 stimulates proinflammatory signaling pathways, which ultimately cause the release of inflammatory cytokines such as TNF‐α and IL‐1β [21, 22]. In addition, peptides that are released during fibrin formation (e.g., fibrinopeptide B) can act as chemoattractants for PMNs [23].

Healthy people have plasma levels of 1.5–3.5 mg/mL of fibrinogen. The expression of fibrinogen increases during an acute phase response, in pregnancy, or due to injuries. This increase in fibrinogen concentration is stimulated, for example, by glucocorticoids and cytokine IL‐6 [24]. In clinical practice, fibrin degradation products such as D‐dimers often serve as indicators of inflammation and increased coagulation activity, and as risk predictors for thrombotic events [25].

### 1.4 Study Plan

**FIGURE 1:**
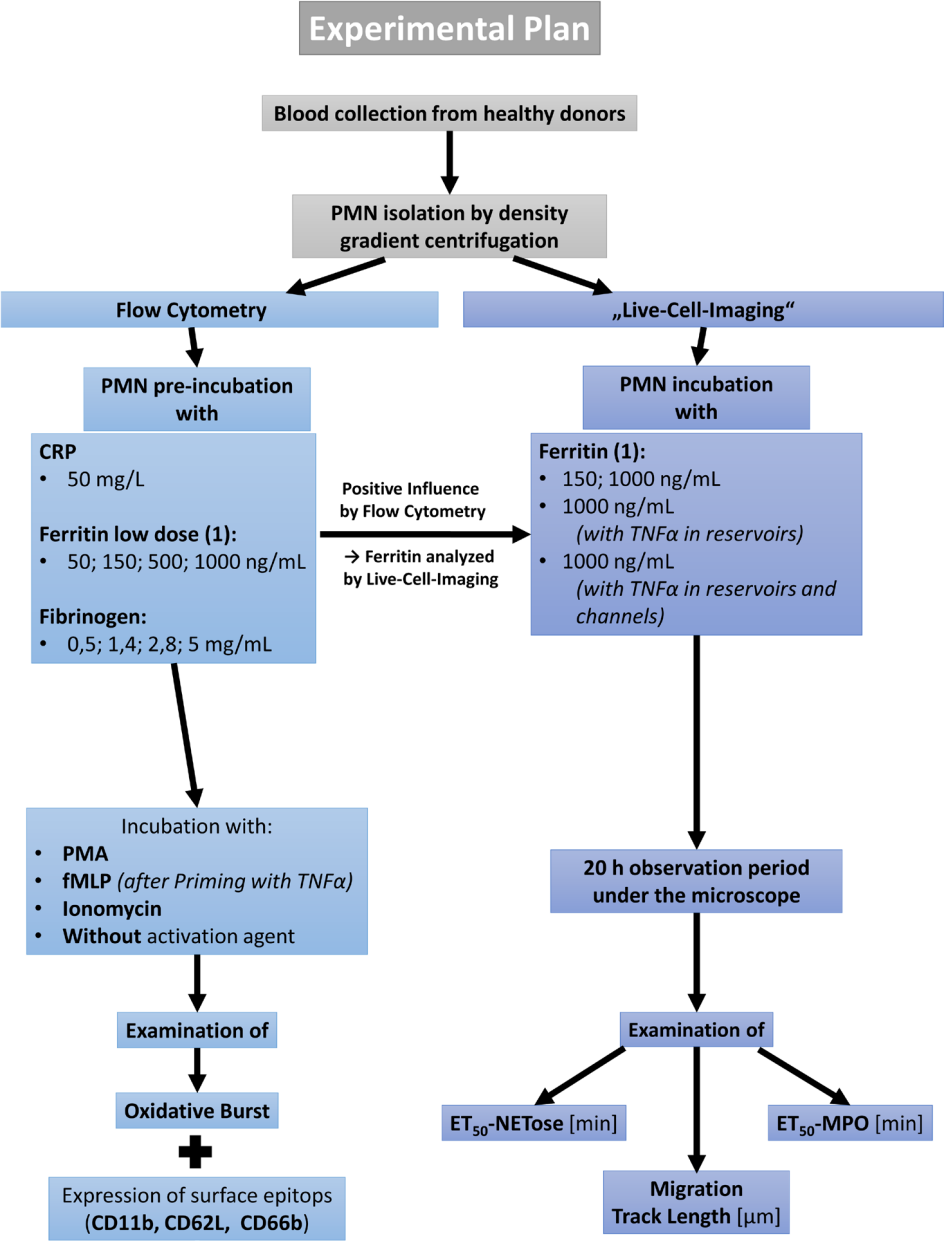
Workflow of the experiments.

In order to investigate a potential influence of APPs on neutrophil functions, PMNs were incubated with the APPs CRP, ferritin, and fibrinogen and then examined by means of flow cytometry and live cell imaging (LCI) using a fluorescence microscope. The experimental plan is shown in Figure 1.

## 2 Materials and Methods

### 2.1 Ethical Considerations

All experiments were conducted in accordance with the Declaration of Helsinki and approved by the local ethics committee of the University of Regensburg (file number 20‐1919‐101).

### 2.2 Sample Collection and Preparation

Whole blood samples were collected from volunteers after informed consent. A safety multifly cannula 0.9 × 19 mm (Sarstedt AG and Co., Nuembrecht, Germany) and an S‐Monovette 7.5 mL lithium heparin (Sarstedt AG and Co.) were used for this purpose.

### 2.3 Density Gradient Centrifugation

PMN cells were isolated from whole blood at room temperature using density gradient centrifugation for 20 min at 756 *g*, passing through PBMC (Lympho) Spin Medium layered over Leuko Spin Medium (both from pluriSelect Life Science, Leipzig, Germany). After different phases were formed due to physical properties, the layer in which PMN cells were located was milky white and located directly above the erythrocyte‐rich phase. The phases above the PMN layer were removed. Then the PMN cells were collected with a pipette and resuspended in Roswell Park Memorial Institute (RPMI)‐1640 culture medium (Pan Biotech, Aidenbach, Germany) and 10% fetal calf serum (Sigma‐Aldrich, St.Louis, MO, USA) at a concentration of 18 × 10^6^ cells/mL, as described before [26, 27].

### 2.4 Flow Cytometry

#### 2.4.1 Cell Activation

The isolated PMN cells were equally divided and incubated with CRP (50 mg/L; Bio‐Techne GmbH, Wiesbaden, Germany), fibrinogen (0.5–5 mg/mL; Sigma‐Aldrich, St.Louis, MO, USA), ferritin (50–1000 ng/mL; Sigma‐Aldrich, St.Louis, MO, USA), or no APP as control for 30 min at 37°C in a water bath. Afterwards, various activating substances were added to the PMNs and incubated for 20 min at 37°C also in a water bath. The activation substances were 10 μL PMA (10 μM, Sigma Aldrich), 35 μL ionomycin (10 μg/mL, Sigma Aldrich), or 10 μL fMLP (10 μM, Sigma‐Aldrich, St.Louis, USA). The samples to be activated with fMLP were primed with TNFα (PeproTech Germany, Hamburg, Germany) for 10 min before incubation (TNFα concentration: 10 ng/mL). Shortly before the measurement, 10 μL propidium iodide (PI, 1.5 mM, Thermo Fisher Scientific, Waltham, USA) was added. For one distinct test series, no activator was used as a control [28].

#### 2.4.2 Detection of the Oxidative Burst

Following pre‐incubation with the APPs and the different activation substances (see above), PMN cells were washed once by adding 2 mL of Dulbecco's Phosphate Buffered Saline (PBS, Sigma Aldrich, Steinheim, Germany) to the samples and centrifugation for 5 min at 1500 rpm. The liquid phase (without cells) was then drained, leaving cells intact. For the detection of the oxidative burst, 1 mL PBS (without CaCl_2_ and MgCl_2_), 10 μL dihydrorhodamine 123 (DHR, 10 μM, Thermo Fisher Scientific, Waltham, USA) and 10 μL seminaphtharhodafluorine (SNARF, 10 μM, Thermo Fisher Scientific, Waltham, USA) were added [27].

#### 2.4.3 Antigen Expression of CD11b, CD62L and CD66b

For immunophenotyping, the expression of various antigens on the surface of PMNs was examined using commercially available antibodies labeled with either phycoerythrin (PE), fluorescein isothiocyanate (FITC), or allophycocyanin (APC), following the manufacturer's protocol. The antibodies used were CD11b PE (ICRF44), CD62L FITC (DREG‐56), and CD66b APC (G10F5), all from BioLegend (San Diego, CA, USA) [27].

#### 2.4.4 Flow Cytometry Measurements

Flow cytometry measurements were performed with a FACS Calibur (“fluorescence‐activated cell sorting”) and CellQuest Pro software version 5.2 (both BD Biosciences, Eremboegem, Belgium) as described by Doblinger et al. [27]. All measurements were analyzed with FlowJo version 10.0.7 (FlowJo LLC, Ashland, Oregon, USA). PMNs were identified based on their size and granularity (forward scatter (FSC)/side scatter (SSC)).

Median fluorescence intensities “arbitrary fluorescence units” [afu] were calculated from the respective dot‐plots. Flow cytometry was used to detect ROS production and the expression of the surface proteins CD11b, CD62L, and CD66b. The PMNs were processed differently to measure the oxidative burst and antigen expression [27].

### 2.5 Chemotaxis Model

#### 2.5.1 Preparation of the μSlide Chemotaxis Chambers

**FIGURE 2:**
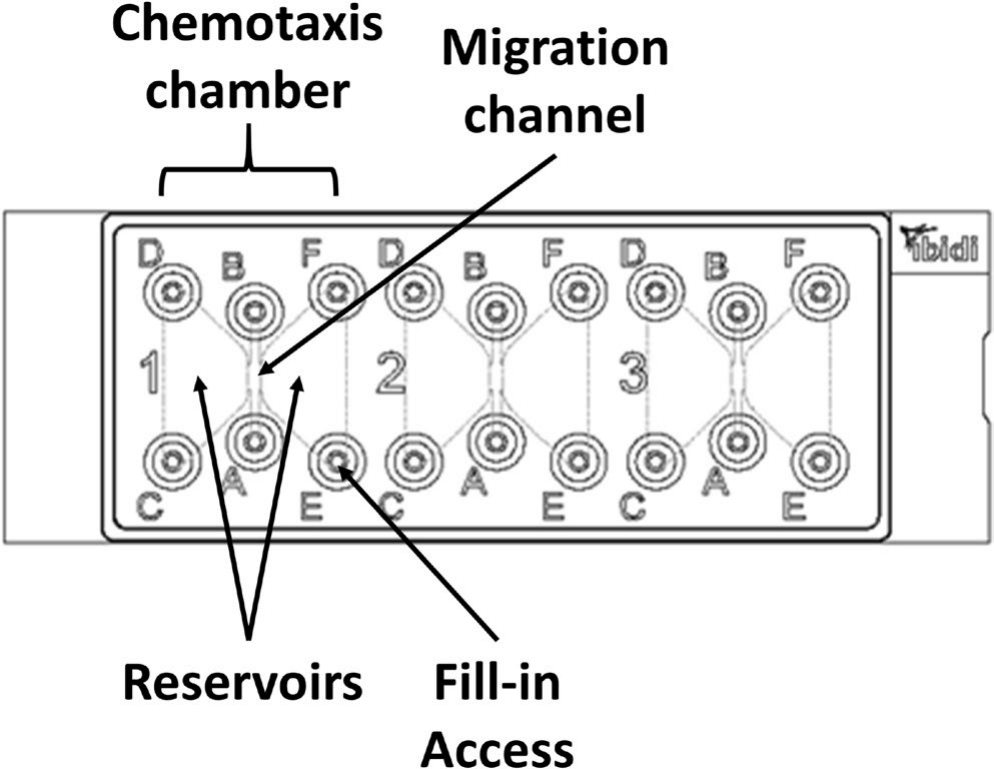
Composition of the IBIDI μSlide chemotaxis chambers [29].

μSlide chemotaxis chambers (IBIDI GmbH) were utilized for live cell imaging. At each of the three positions, these chambers feature a central channel and 2 reservoirs on both sides (see Figure 2).

Each channel was filled with 6.5 μL matrix. This matrix was taken from a mixture consisting of 17 μL cells (with 18 × 10^6^ cells/mL; isolation see Section 2.3), 18–33 μL nutrient medium (including anti‐MPO‐APC and DAPI), 50 μL type I collagen solution (PureCol Type I Bovine Collagen, Advanced BioMatrix, Carlsbad, USA) and 5–15 μL of ferritin (150–1000 ng/mL, Sigma Aldrich, St.Louis, MO, USA as described before [26]). In some experiments, TNFα (final concentration in the channel: 10 ng/mL) was also added as an additional activating substance.

In total, *n* = 11 LCI experiments were conducted involving the incubation of PMNs with ferritin. The conditions of the individual experiments varied, particularly regarding the addition of TNFα. fMLP was consistently added as a chemoattractant to one of the reservoirs. At first, *n* = 4 experiments were carried out with TNFα exclusively in the reservoirs of the IBIDI μSlide. Further on, *n* = 7 experiments were performed with TNFα in the channels and reservoirs of the IBIDI μSlide (details are shown in Table 1).

**TABLE 1:** Overview of experimental conditions “live cell imaging” experiments with ferritin and TNFα.

	Control	Group 1	Group 2	Group 3
TNFα (ng/mL)	0	10	0	10
Ferritin (ng/mL)	0	0	1000	1000

	Control	Group 4	Group 5
TNFα (ng/mL) (channel + reservoirs)	0	10	10
Ferritin (ng/mL)	0	0	1000

#### 2.5.2 Microscopy and Live Cell Imaging

For live cell imaging, the prepared μSlides were examined using a Leica DMi8 microscope, a Leica DFC9000 camera, and a pE‐4000 light source (all: Leica Microscopy and Systems GmbH, Wetzlar, Germany, as described before [26]).

Live cell imaging focused on the investigation of various PMN functions: migration behavior, MPO release (ET_50_MPO) as well as NET formation (ET_50_NETosis) was investigated. MPO release was assessed by means of 0.5 μg/mL anti‐MPO‐APC antibodies (Miltenyi Biotech). NETosis was detected using 4′,6′‐diamidino‐2‐phenylindole dihydrochloride (DAPI, Sigma‐Aldrich Chemie GmbH, Steinheim, Germany). After microscope start, photos were taken every 45 s in phase contrast and in the fluorescence channels for 20 h. Control of the microscope and the observation process was computer‐aided using the Application Suite X software platform (version 3.4.2.18368).

#### 2.5.3 Analysis of the Image Data

**FIGURE 3:**
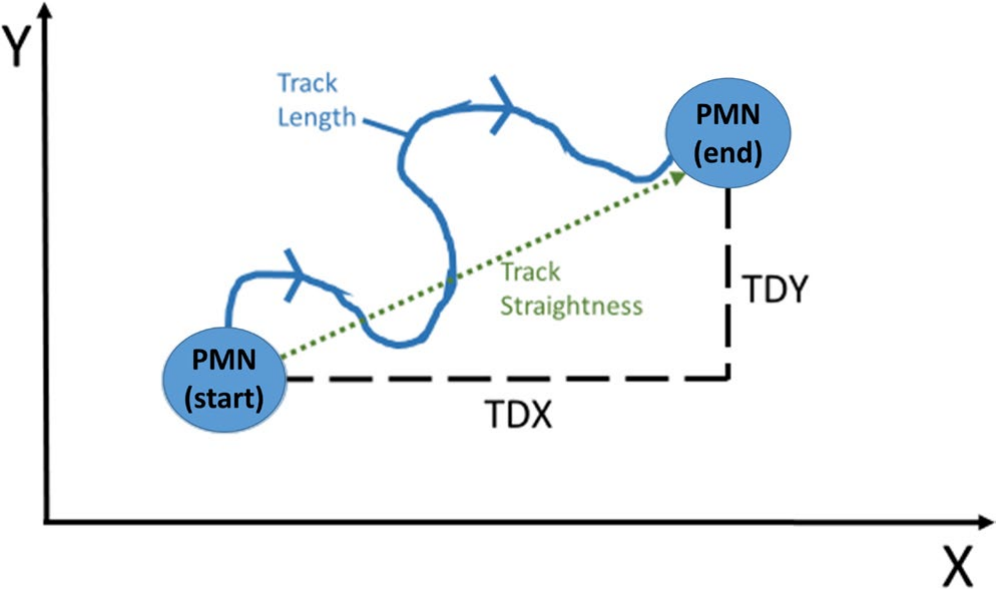
Illustration of the migration parameters: TDX, Track Displacement Length X; TDY; Track Displacement Length Y within the set cartesian coordinate system; TL, track length.

The image series generated by the microscope (a total of 1600 images per channel during 20 h observation time) were analyzed with the Imaris 9.0.2 software (Bitplane, Zurich, Switzerland). Cell migration was analyzed in phase contrast. To quantify migration, axes in the x and y directions were established for each channel, similar to a Cartesian coordinate system, covering the entire length of the analyzed image area (see Figure 3). The following parameters were recorded: Track Length (TL), which indicates the total migration length of a PMN cell, Track Displacement Length, which indicates the Euclidean distance, and Track Straightness (indicates TDL/TL). Moreover, Track Displacement Length X (TDX) and Track Displacement Length Y (TDY), which only describe the proportion of migration of the cell in the X or Y direction, and track duration (TD), which indicates the time that a PMN cell migrated, were detected. The results of the migration analysis were subdivided into 30 min observation intervals (time slots). Filters for track length > 25 μm (track length) and track duration > 15 min were set to exclude false double detection.

All dyes utilized for measuring MPO and NETosis were visible in the fluorescence images as colored regions. The software IMARIS recognized these areas in each image semi‐automatically and exported the data to a separate Excel file, which included the areas [μm^2^] for each specific color in every image. All areas captured at the same time point were compiled in Excel [26, 27].

**FIGURE 4:**
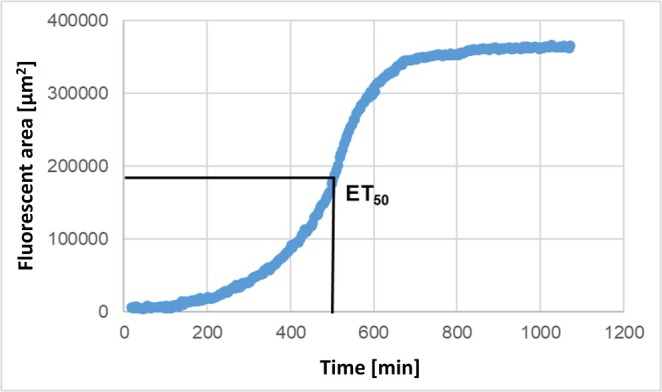
Example graph ET_50_ value for determining NETosis and MPO release.

NETosis assessment with DAPI and anti‐MPO yielded sigmoidal curves (see Figure 4). The time to half‐maximum effect, ET_50_, was calculated using Phoenix 64 Version 8.0.0 (Certara Inc., New York, USA). ET_50_ values that indicate time points beyond the duration of the test were excluded.

### 2.6 Statistical Analysis

SPSS Statistics (version 25 and version 26, IBM, Armonk, USA) was used for statistical analysis. Only non‐parametric tests (Mann–Whitney *U* or Kruskal–Wallis) were used for better comparability of the various results and due to the small number of values in some cases. The data were graphically summarized in figures as boxplots, indicating the interquartile ranges. A line graph with error bars indicating a 95% confidence interval (CI) is used to illustrate the results and, in particular, the temporal course of the intensity of ROS production more clearly. *p*‐values < 0.05 were considered statistically significant. No data were excluded.

## 3 Results

### 3.1 Characteristics of the Test Persons

Experiments were performed with blood from 11 healthy donors. Their characteristics are summarized in Table 2 and listed in detail in Table S1 in the supplement.

**TABLE 2:** Properties of the granulocyte donors.

Characteristics	Value
Number of experiments (*n*)	11
Male/female	7/4
Age [a]	29 (20–56)
Height [cm]	178 (168–194)
Weight [kg]	71 (50–91)

### 3.2 Flow Cytometric Measurements

#### 3.2.1 Influence of CRP

**FIGURE 5:**
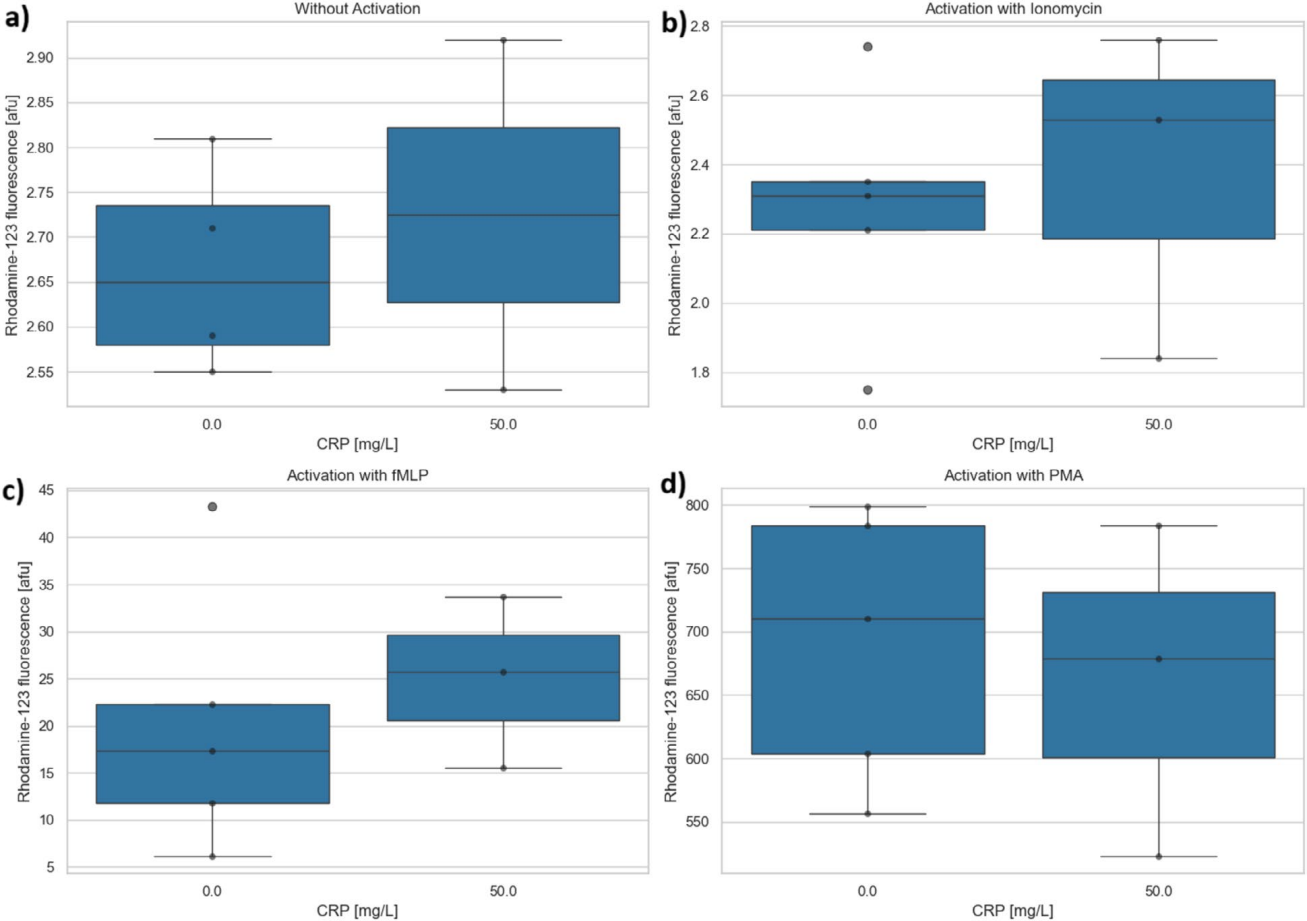
Rhodamine‐123 fluorescence after preincubation without or with CRP 50 mg/L (a) without activation or subsequent activation with (b) ionomycin, (c) TNFα/fMLP or (d) PMA.

**FIGURE 6:**
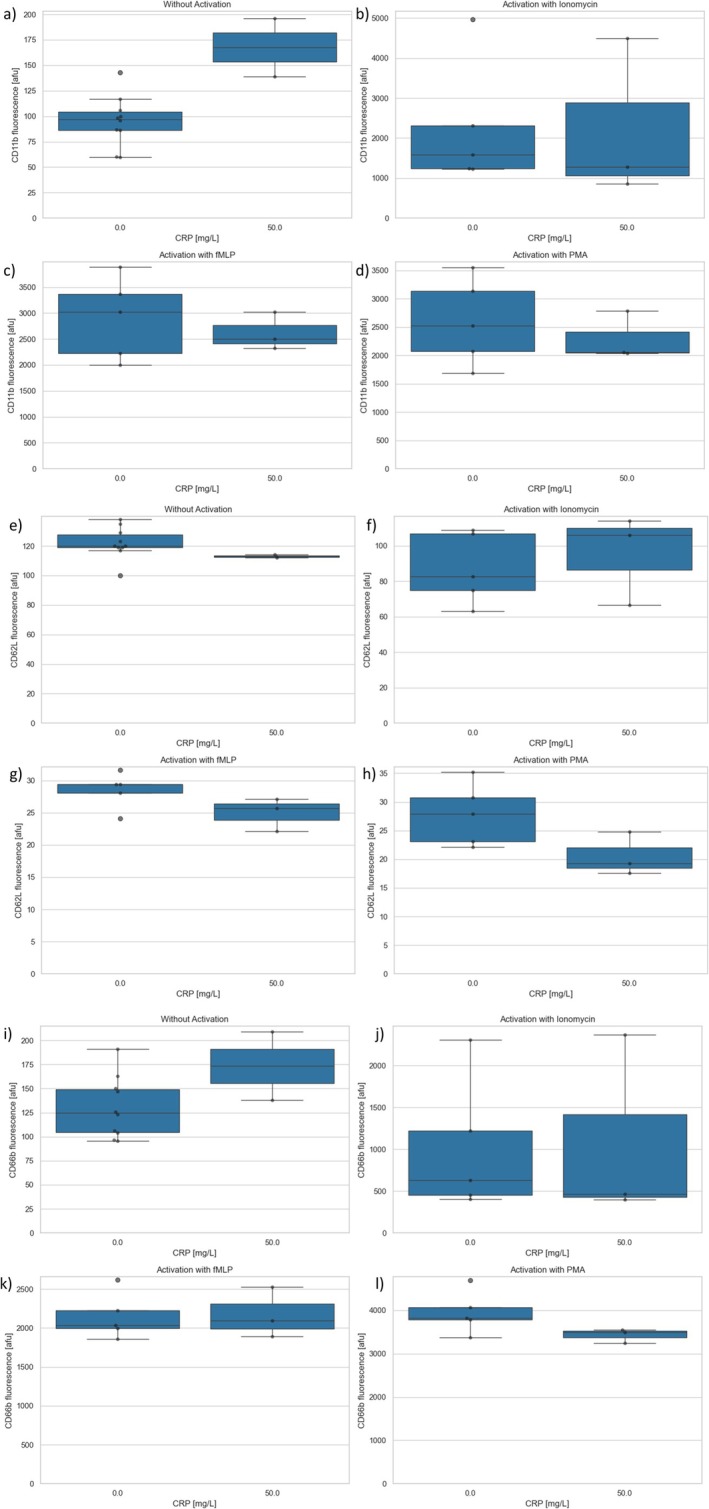
(a–d) CD11b expression after CRP preincubation (a) without activation and activation with (b) ionomycin (c) TNFα/fMLP (d) PMA. (e–h) CD62L expression after CRP preincubation (e) without activation and activation with (f) ionomycin (g) TNFα/fMLP (h) PMA (i–l) CD66b expression after CRP preincubation (i) without activation and activation with (j) ionomycin (k) TNFα/fMLP (l) PMA.

A total of *n* = 3 experiments were performed using flow cytometry after preincubation of the PMNs with CRP (50 mg/L) or control. Whereas there were partly large variations due to the different activation substances, no change in the oxidative burst due to preincubation of the PMNs with CRP could be observed compared to control (Figure 5). Furthermore, no increased expression of CD11b, CD62L, and CD66b could be detected (Figure 6).

#### 3.2.2 Influence of Ferritin

**FIGURE 7:**
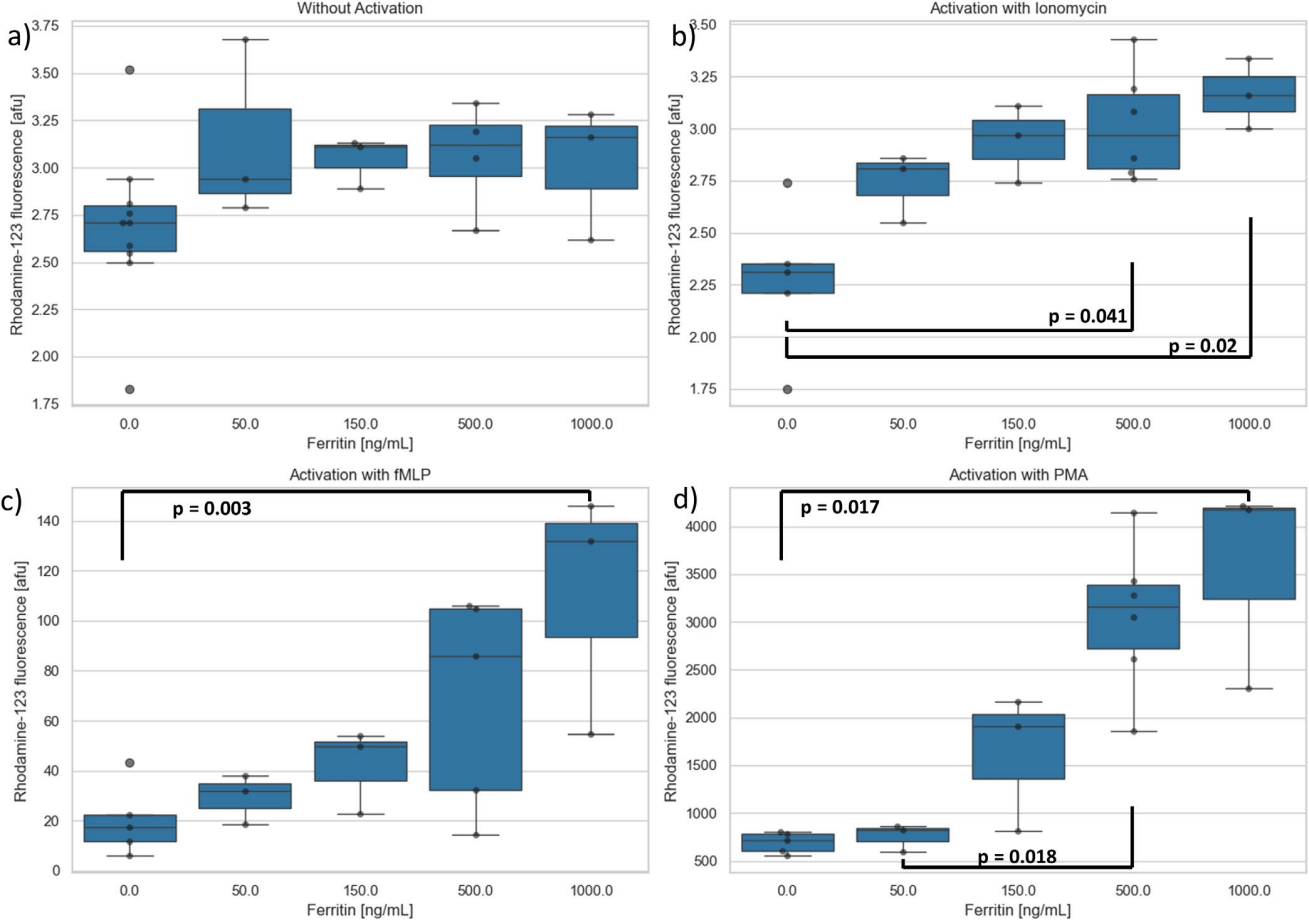
Rhodamine‐123 fluorescence after preincubation with different concentrations of ferritin and (a) without activation or subsequent activation with (b) ionomycin, (c) TNFα/fMLP or (d) PMA.

A total of *n* = 4 experiments were performed using flow cytometry after preincubation of the PMNs with ferritin or control. Without activation, Rhodamine‐123 fluorescence as a marker of PMN oxidative burst was between 2.65 afu (0 ng/mL ferritin) and 3.16 afu (1000 ng/mL ferritin), independent of ferritin concentration. After activation with ionomycin, TNFα/fMLP, or PMA, Rhodamine‐123 fluorescence increased continuously with rising ferritin concentrations, the differences becoming statistically significant at 500 ng/mL (ionomycin, PMA) or 1000 ng/mL of ferritin (TNFα/fMLP) (Figure 7).

**FIGURE 8:**
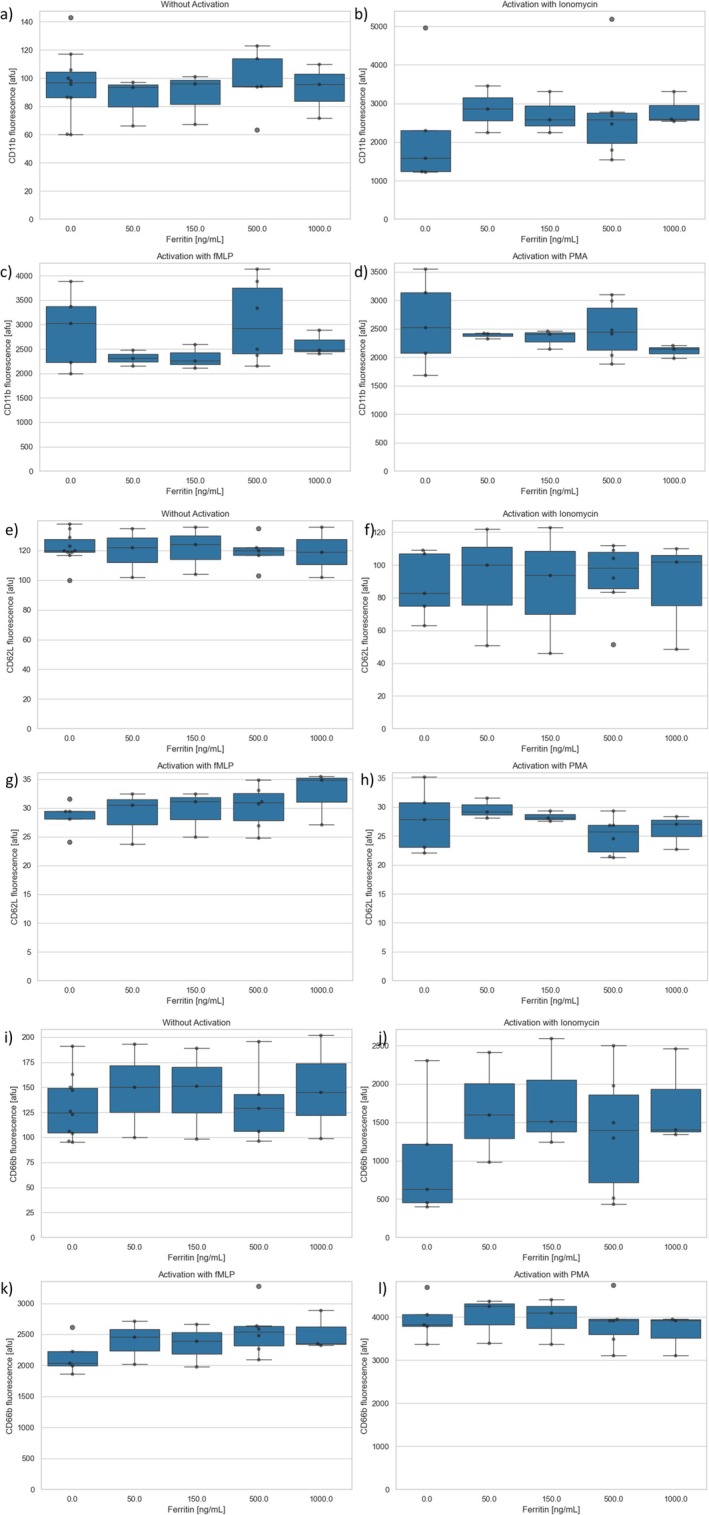
(a–d) CD11b expression after ferritin preincubation (a) without activation and activation with (b) ionomycin (c) TNFα/fMLP (d) PMA. (e–h) CD62L expression after ferritin preincubation (e) without activation and activation with (f) ionomycin (g) TNFα/fMLP (h) PMA (i–l) CD66b expression after ferritin preincubation (i) without activation and activation with (j) ionomycin (k) TNFα/fMLP (l) PMA.

No significant changes in the expression of the surface antigens CD11b, CD62L, or CD66b by ferritin were detected (*p* > 0.05) regardless of the activating substance (see Figure 8).

#### 3.2.3 Influence of Fibrinogen

**FIGURE 9:**
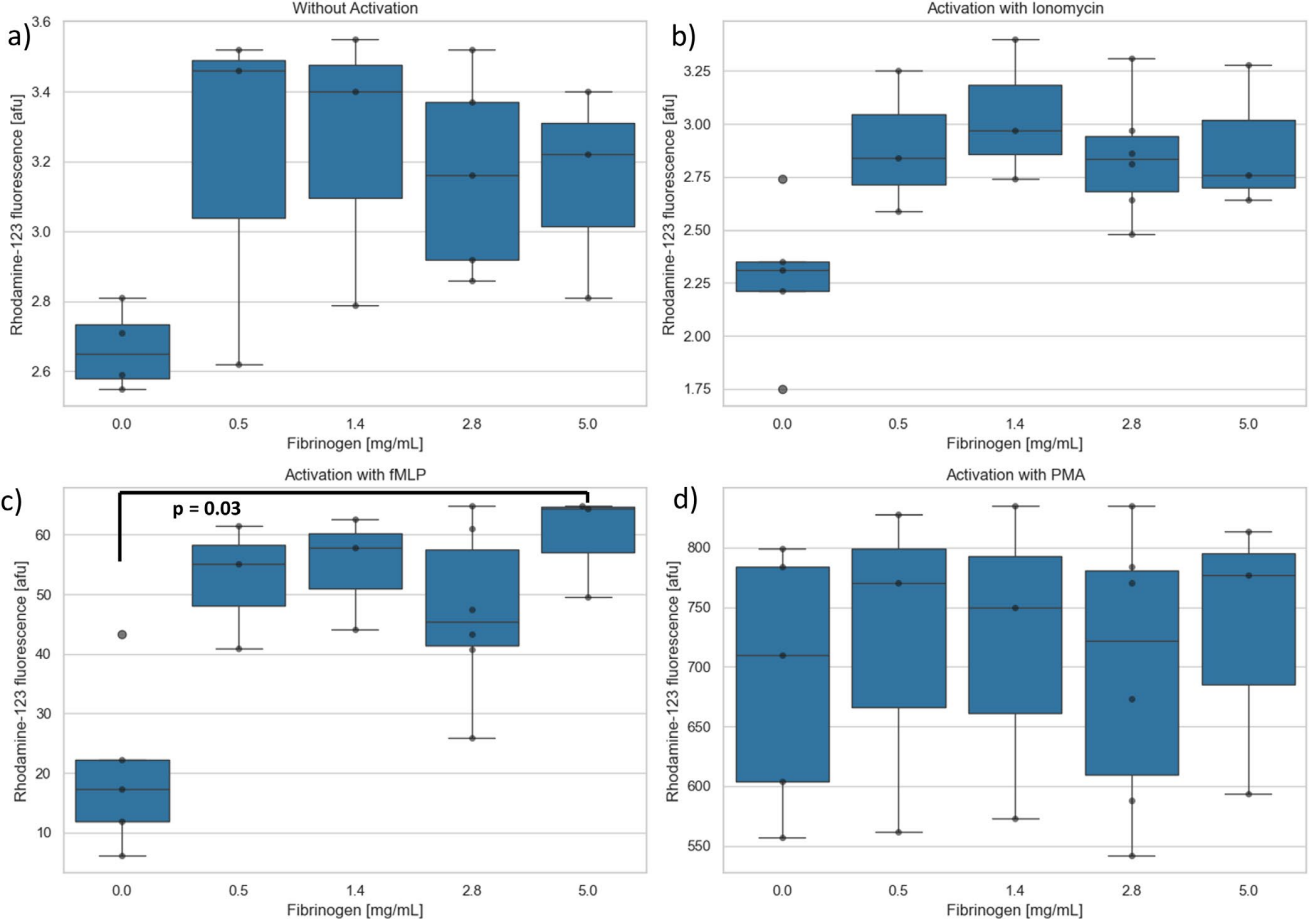
Rhodamine‐123 fluorescence after preincubation with different concentrations of fibrinogen (a) without activation and subsequent activation with (b) ionomycin (c) TNFα/fMLP or (d) PMA.

A total of *n* = 4 experiments were performed using flow cytometry after preincubation of the PMNs with fibrinogen or control. No significant changes in the oxidative burst could be observed with the activating substances Ionomycin and PMA, as well as without activating substances, regardless of the fibrinogen concentration (Figure 9a,b,d). When PMNs were preincubated with fibrinogen and subsequently activated with TNFα/fMLP, the oxidative burst was higher for each concentration of fibrinogen compared to control (afu = 17), the difference reaching significance at the highest tested concentration (Figure 9c).

**FIGURE 10:**
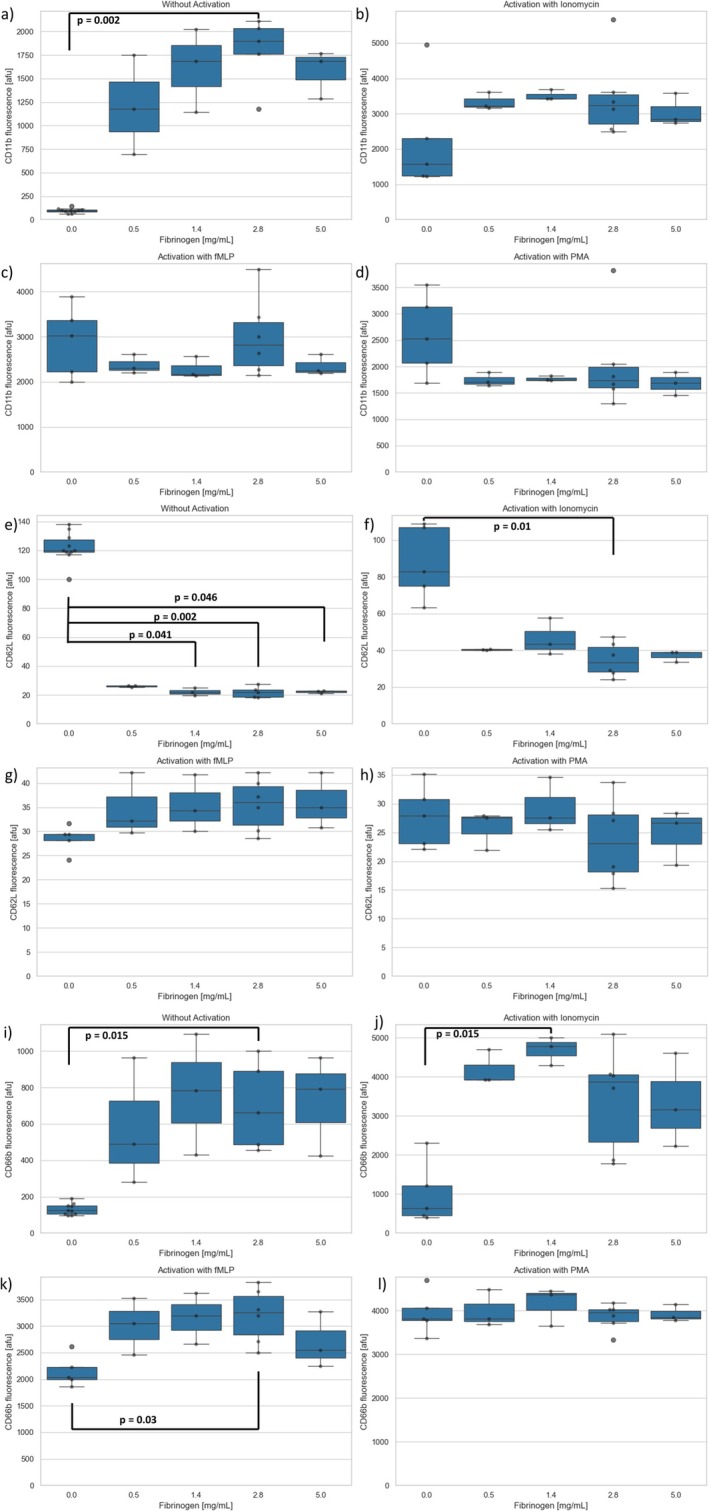
(a–d) CD11b expression after fibrinogen preincubation (a) without activation and activation with (b) ionomycin (c) TNFα/fMLP (d) PMA. (e–h) CD62L expression after fibrinogen preincubation (e) without activation and activation with (f) ionomycin (g) TNFα/fMLP (h) PMA (i–l) CD66b expression after fibrinogen preincubation (i) without activation and activation with (j) ionomycin (k) TNFα/fMLP (l) PMA.

A concentration‐dependent significant increase due to fibrinogen in the median intensity of CD11b was noted without further activation (Figure 10a). No differences were observed after activation with ionomycin, TNFα/fMLP, or PMA (Figure 10c,d). CD62L expression decreased significantly without further activation and with ionomycin activation after pre‐incubation with fibrinogen (Figure 10e,f), but not when activated with TNFα/fMLP or PMA (Figure 10g,h). CD66b increased significantly with increasing fibrinogen concentration without activation and after activation with ionomycin or TNFα/fMLP (Figure 10i–k), but not with PMA (Figure 10l).

### 3.3 Results of the Live Cell Imaging Experiments

#### 3.3.1 Results of the LCI‐Experiments With Ferritin

##### 3.3.1.1 Chemotaxis

**FIGURE 11:**
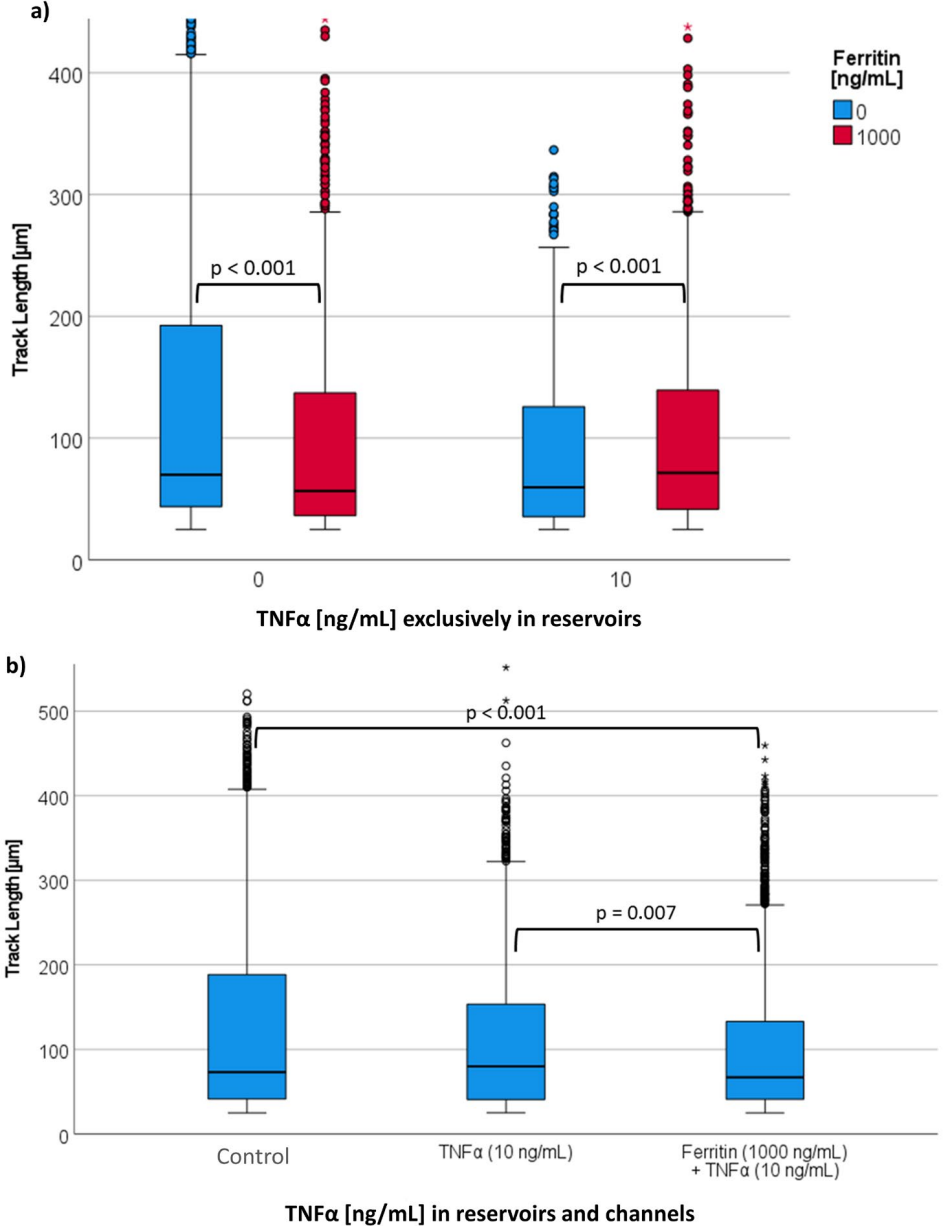
(a) Results of parameter TL under the influence of ferritin (0, 1000 ng/mL) and TNFα (0, 10 ng/mL) in the reservoirs in the first hour of the observation period. The number of cells observed per boxplot was between 560 and 1302. (b) Results of parameter TL under the influence of ferritin (0, 1000 ng/mL), as well as TNFα (0, 10 ng/mL) in the channel and the reservoirs in the first hour of the observation period. The number of cells observed per boxplot was between 1977 and 2277.

The evaluation of the PMN migration after activation by TNFα in the reservoirs is shown in Figure 11a. For a better overview, the results of the first hour of observation are summarized here. Without TNFα, the addition of ferritin reduced the track length from 70 to 57 μm. In contrast, in the presence of TNFα, ferritin increased the track length from 60 μm to 72 μm.

The evaluation of the PMN migration of the experiments after activation by TNFα in the reservoirs and channels is shown in Figure 11b. The presence of TNFα reduced TL from 80 μm to 73 μm. Ferritin further reduced TL from 73 μm to 67 μm (see Figure 11b).

##### 3.3.1.2 Results of Neutrophil ROS Production, MPO Release and NETosis

**FIGURE 12:**
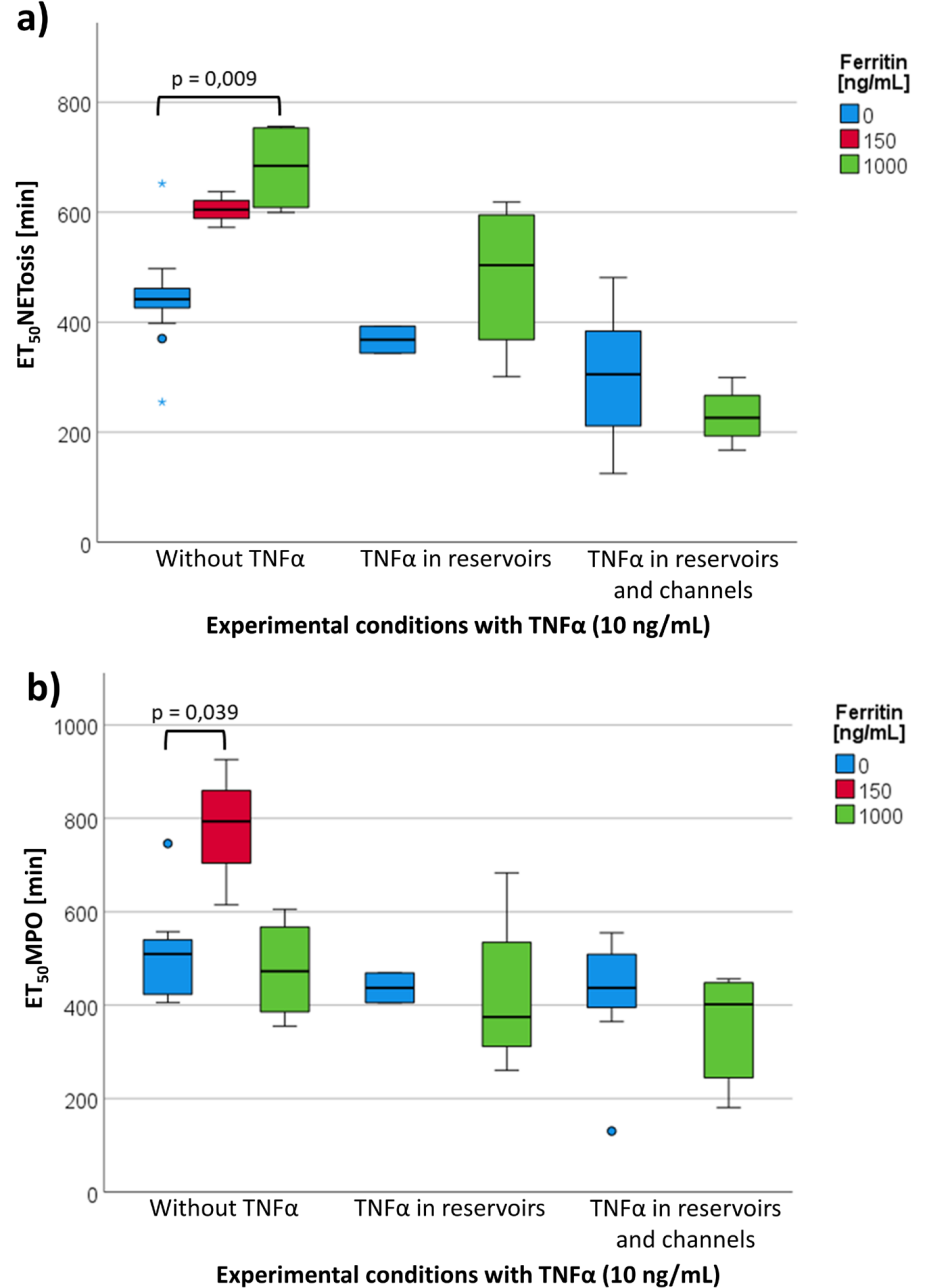
(a) ET_50_NETosis under the influence of ferritin (150; 1000 ng/mL) and TNFα (10 ng/mL). *N* was between 2 and 14 per boxplot. (b) ET_50_MPO of MPO release under the influence of ferritin (150; 1000 ng/mL) and TNFα (10 ng/mL). *N* was between 2 and 14 per boxplot.

Without TNFα, a concentration‐dependent increase of ET_50_NETosis by ferritin from 442 to 685 min was observed, whereas no significant effect was observed in the presence of TNFα (Figure 12a).

Without TNFα, MPO release was earlier without (ET_50_MPO = 510 min) than with 150 ng/mL ferritin (ET_50_MPO = 793 min). No other statistically significant differences were found, regardless of whether TNFα was present in the reservoirs or additionally in the channels (see Figure 12b).

## 4 Discussion

In this study, PMNs were isolated by density gradient centrifugation and then incubated with various APPs and immune activators. A panel of PMN functions was quantified and analyzed by means of flow cytometry. These functionalities included the expression of the surface proteins CD11b, CD62L, and CD66b, chemotaxis, the oxidative burst, MPO release, and NETosis.

### 4.1 Influence of CRP on Neutrophil Granulocytes

In order to investigate the effect of CRP on PMNs, PMNs were incubated with 50 mg/L of CRP, and then analyzed by flow cytometry. This concentration and the particularity of conducting tests also without activation substances were based on various statements in the literature. Ling et al. mainly detected a reduction of the intra‐ and extracellular ROS production of PMNs under the influence of human plasma CRP. However, this was dependent on whether the PMNs were stimulated, and if so, which receptor (e.g., FcγR or TLR2/4) was stimulated [30]. Moreover, only monomeric (mCRP) but not native pentameric (nCRP) CRP showed an influence on PMN surface expression [31]. Vulesevic et al. incubated isolated neutrophils for 60 min with 1, 5, and 10 mg/L of homopentameric recombinant human (rh)CRP, and subsequently captured NETs stained with SYTOX Green using confocal microscopy. In the process, the authors described a significant increase (by a factor of 3.5) in NETosis after the treatment of PMNs with 10 mg/L of CRP compared to the PMNs that were exposed to 1 mg/L of CRP. Ultimately, rhCRP (10 mg/L) was characterized as being an equally effective trigger of NETosis as IL‐8 (25 nM) [32], which has been known to be an efficient trigger of NET formation already since 2004 [33].

Since the highest concentration to date (in literature) of 10 mg/L of CRP only corresponds to a slightly increased pathological concentration (normal range < 5 mg/L, typical concentrations in severe bacterial infections 100–500 mg/L), flow cytometry was used to collect further data generated after PMN incubation with 50 mg/L of CRP. This served to verify the influence of a higher pathological CRP concentration on the PMNs. After incubation with 50 mg/L of CRP, PMNs were additionally activated with TNF‐α/fMLP, PMA, or ionomycin. These flow cytometry tests showed no effect of CRP on the antigen expression or the oxidative burst.

### 4.2 Influence of Ferritin on Neutrophil Granulocytes

Ferritin was chosen as an acute phase protein (APP) for this study because while there is no evidence of a direct influence of ferritin on PMNs in the literature, there is a clinical correlation between increased PMN numbers, increased MAC‐1 expression, increased oxidative stress, and increased ferritin values [34]. In our experiments, ferritin led to a strong concentration‐dependent increase in oxidative burst. Thus, a direct correlation between the oxidative stress level in the body and the ferritin level is quite possible. However, ferritin had no influence on the antigen expression of PMNs in our experiments (see Figure 8).

Due to the significant results in the flow cytometry tests regarding the oxidative burst, live cell imaging tests were also performed with ferritin. First, PMNs were incubated for 30 min with 0, 150, or 1000 ng/mL of ferritin. By default, fMLP was added to one of the reservoirs. Initially, TNF‐α was not part of the test series. These conditions led to a delayed NETosis of PMNs by 1000 ng/mL of ferritin. Due to the strong ROS production of PMNs after the incubation with ferritin in the flow cytometry tests, a premature NETosis would rather have been expected. Therefore, further tests were conducted with TNF‐α. And indeed, track lengths showed statistically significant differences. With regard to MPO release and NETosis capacity, there were no changes that could be attributed exclusively to ferritin but were probably due to the effect of TNF‐α on PMNs.

Nevertheless, oxidative stress is a pivotal point in the pathophysiology of life‐threatening conditions like sepsis or other hyperinflammation syndromes. There is an association between immune dysregulation and oxidative imbalance. Smail et al. observed that biomarkers like the neutrophil‐lymphocyte ratio (NLR) and ferritin were predictors of the severity and mortality of COVID‐19 [35]. It is conceivable that the pro‐inflammatory influence of ferritin on PMNs is a central connection in pathophysiology.

### 4.3 Influence of Fibrinogen on Neutrophil Granulocytes

The effect of various concentrations (0.5, 1.4, 2.8, 5 mg/mL) of fibrinogen on PMNs was examined by means of flow cytometry. The concentration of 2.8 mg/mL was taken from the literature, where a delayed NETosis could be demonstrated after activation of the PMNs with ionomycin [36]. As a result of the tests, an increasing effect of fibrinogen on the oxidative burst was detected after activation with TNF‐α/fMLP, but not after activation with PMA or ionomycin. Han et al. recently reported an effect of PMNs on fibrinogen, but not vice versa. According to that, the ROS production of PMNs is co‐responsible for the oxidation and subsequent degradation of fibrinogen [37]. Looking at the data on the changes in antigen expression, a significant increase in CD11b expression can be seen after sole stimulation with fibrinogen. There was also an increased expression of CD66b both after preincubation with fibrinogen without activating substance and after additional activation by TNF‐α/fMLP and ionomycin. This change has already been demonstrated by Rubel et al. They described that the binding of fibrinogen to CD11b triggers the activation of PMNs by a CD11b‐dependent mechanism, which results in an increase in intracellular Ca^2+^, upregulation of CD11b, and degranulation [38]. Moreover, this CD11b‐dependent binding resulted in increased expression of CD66b, which is maximal at 60 min after incubation with 2 mg/mL of human fibrinogen [38]. The expression of CD62L was inhibited after preincubation with fibrinogen, both without activating substance and with subsequent activation by ionomycin. This effect has not been described in the literature, but since the expression of CD11b, as well as of CD66b, was changed, and this change was also known, it is likely that the expression of CD62L was also influenced by fibrinogen in a CD11b‐dependent manner.

If we take a closer look at the tasks of the individual surface epitopes and place them in the context of the changes caused by fibrinogen, we can see that fibrinogen appears to activate the PMNs: Upregulation of CD11b allows activated PMNs to attach to the vascular endothelium and start the process of transmigration through blood vessel walls. CD62L is one of the first adhesion molecules on PMNs that interact with the endothelium in the bloodstream and is crucial for their transmigration through blood vessels and therefore is upregulated in the blood [39]. CD66b, a receptor specific to granulocytes and part of the carcinoembryonic antigen family, primarily facilitates phagocytosis [40].

### 4.4 Discussion of the Results in Context of Other APP

The data from our study show different effects of the APP on PMNs, some activating, some non‐activating. Our data are therefore consistent with earlier studies, which also showed that APP does not necessarily have a PMN activating effect. Haptoglobin, for example, not only activates PMNs at extravascular sites, but actively participates in all the processes from PMN recruitment and free radical quenching to tissue repair and regeneration, thereby performing an antioxidant effect [41]. Alpha‐1‐antitrypsin inhibits PMN ROS production and serine proteases and suppresses LPS‐induced IL‐1β, IL‐8, and TNF‐α release from PMNs [42].

In contrast, Serum Amyloid A (SAA) participates in enhancing PMN chemotaxis to inflamed tissues and inducing directional migration of PMN [43]. Stimulation of PMN by SAA resulted in a rapid and transient increase of cytosolic calcium concentration and up‐regulation of cell‐surface expression of antigens involved in adhesion and microbial recognition such as CD11c and CD16 probably mediated by the formyl peptide receptor 2 (FPR2) [44, 45].

Nevertheless, PMNs are also able to degrade APP, e.g., SAA. Alteration in SAA proteolysis by activated PMNs may contribute to the deposition of amyloid fibrils in the tissues of patients with chronic inflammatory disease [46].

The crucial role of PMNs in innate immunity, combined with their propensity to induce tissue damage, necessitates the precise and tightly regulated control of PMN activity [39]. The various pro‐ and anti‐inflammatory effects of the different acute phase proteins are very likely an expression of the body's attempt to activate the neutrophil immune response quickly and safely, to prevent an excessive reaction, and to deploy it as precisely as possible.

### 4.5 Limitations

The use of PMNs from a cohort of only 11 healthy volunteers represents a relatively limited sample size. This, as well as the variability in donor responses, requires explicit discussion in order to appropriately contextualize the results.

When performing single‐cell analysis with PMNs, the question raises whether to consider each PMN cell on its own (i.e., as a single unit), or to consider the PMN cells of one single donor as a single unit, aggregating the individual cells to the level of the donor. This also includes the question of which difference is greater: the difference between the individual cells or the inter‐individual difference between the donors.

As our sample size is relatively small, we ensured that no donor was duplicated. This was to prevent technical replicates (i.e., multiple neutrophil cells from the same donor) from being represented as biological replicates, which would have posed a problem in terms of statistical independence [27, 47]. Our considerations are based on a publication of Grieshaber‐Bouyer et al. who discovered through genetic analyses in 2021 that PMNs represent a single developmental continuum [48].

The methodology is limited in its representation of reality. Many things happen very differently within the human body than they do in vitro. Granulocytes are considered to be very sensitive cells that do not survive long outside the human body (8 h to 5.4 days) [49]. Thus, there are research groups that isolate PMNs without centrifugation because this probably already leads to the activation of the PMNs [50, 51]. Hundhammer et al. demonstrated, for example, that increased g‐force and duration of centrifugation lead to decreased functionality (e.g., reduced migration behavior and oxidative burst) of PMNs [52]. On the other hand, the centrifugation of granulocytes is a common method of isolation that many research groups use for their work [53, 54, 55, 56]. As we could not currently rule out the influence of age and sex of the PMN donors (unpublished data indicate an influence of both), the selection of the PMN donors was balanced in both criteria for our experiments. Nonetheless, we cannot rule out a bias regarding this aspect. Moreover, neutrophil function in vivo is multifaceted and woven into a complex system of interactions, not only with other components of the immune system. Obviously, our experiments capture only a limited range of these aspects, which we consider to be valid and relevant.

## 5 Summary

This study showed that CRP, ferritin, and fibrinogen exert a moderate influence on neutrophil functionality. In flow cytometric experiments, CRP had no significant effect on PMN function, even though a certain non‐significant activating effect was observed. Ferritin led to a moderate and dose‐dependent increase in ROS production, especially after activation with TNF‐α/fMLP, PMA, or ionomycin. Fibrinogen mainly influenced the expression of the antigens CD11b, CD62L, and CD66b. In the live cell imaging experiment, ferritin reduced PMN migration without TNFα and enhanced PMN migration in the presence of TNFα. Without TNFα, ferritin prolonged NETosis and had a certain dose‐specific effect on MPO release. ROS production was earlier, and ROS intensity was highest in samples with ferritin + TNFα.

These effects on the PMNs were all moderate and showed certain tendencies. Extreme influences could not be detected. Nevertheless, the different APPs appear to have different influences on the PMNs. Future studies should therefore focus on neutrophil intracellular signaling pathways that are activated upon incubation with APP. The determination of gene expression profiles of PMNs after incubation with the different stimuli could be helpful. This could further explain the different PMN behavior after incubation with APPs and elucidate molecular mechanisms underlying the observed changes in neutrophil function.

## Author Contributions

M.A.G., I.W., and M.G.K. designed the experimental plan. M.A.G. and I.W. performed the experiments. R.F.K., I.W., M.A.G., and M.G.K. performed the statistical analysis and interpreted the data. R.F.K. drafted the initial manuscript. All authors provided critical feedback and approved the final manuscript.

## Conflicts of Interest

The authors declare no conflicts of interest.

## Supporting information


**Table S1:** Demographic data of the healthy PMN donors for the live cell imaging.

## Data Availability

The data that support the findings of this study are available on request from the corresponding author. The data are not publicly available due to privacy or ethical restrictions.

## References

[1] H.‐C. Pape , A. Kurtz , and S. Silbernagel , Hrsg Physiologie, vol. 7 (Auflage: Thieme, 2014).

[2] P. J. Delves and I. M. Roitt , “The Immune System. First of Two Parts,” New England Journal of Medicine 343, no. 1 (2000): 37–49. 10882768 10.1056/NEJM200007063430107

[3] C. Gabay and I. Kushner , “Acute‐Phase Proteins and Other Systemic Responses to Inflammation,” New England Journal of Medicine 340, no. 6 (1999): 448–454. 9971870 10.1056/NEJM199902113400607

[4] W. S. Tillett and T. Francis , “Serological Reactions in Pneumonia With A Non‐Protein Somatic Fraction of Pneumococcus,” Journal of Experimental Medicine 52, no. 4 (1930): 561–571. 19869788 10.1084/jem.52.4.561 PMC2131884

[5] Z. Yao , Y. Zhang , and H. Wu , “Regulation of C‐Reactive Protein Conformation in Inflammation,” Inflammation Research 68, no. 10 (2019): 815–823. 31312858 10.1007/s00011-019-01269-1

[6] S. Black , I. Kushner , and D. Samols , “C‐Reactive Protein,” Journal of Biological Chemistry 279, no. 47 (2004): 48487–48490. 15337754 10.1074/jbc.R400025200

[7] D. Bharadwaj , M. P. Stein , M. Volzer , C. Mold , and T. W. du Clos , “The Major Receptor for C‐Reactive Protein on Leukocytes Is Fcgamma Receptor II,” Journal of Experimental Medicine 190, no. 4 (1999): 585–590. 10449529 10.1084/jem.190.4.585 PMC2195602

[8] L. L. Marnell , C. Mold , M. A. Volzer , R. W. Burlingame , and T. W. du Clos , “C‐Reactive Protein Binds to Fc Gamma RI in Transfected COS Cells,” Journal of Immunology 155, no. 4 (1995): 2185–2193. 7636267

[9] S. Chakraborti and P. Chakrabarti , “Self‐Assembly of Ferritin: Structure, Biological Function and Potential Applications in Nanotechnology,” Advances in Experimental Medicine and Biology 1174 (2019): 313–329. 31713204 10.1007/978-981-13-9791-2_10

[10] B. Clyne and J. S. Olshaker , “The C‐Reactive Protein,” Journal of Emergency Medicine 17, no. 6 (1999): 1019–1025. 10595891 10.1016/s0736-4679(99)00135-3

[11] P. Arosio , R. Ingrassia , and P. Cavadini , “Ferritins: A Family of Molecules for Iron Storage, Antioxidation and More,” Biochimica et Biophysica Acta 1790, no. 7 (2009): 589–599. 18929623 10.1016/j.bbagen.2008.09.004

[12] J. Giemza‐Stokłosa , M. A. Islam , and P. J. Kotyla , “Hyperferritinaemia: An Iron Sword of Autoimmunity,” Current Pharmaceutical Design 25, no. 27 (2019): 2909–2918. 31686632 10.2174/1381612825666190709202804

[13] F. M. Torti and S. V. Torti , “Regulation of Ferritin Genes and Protein,” Blood 99, no. 10 (2002): 3505–3516. 11986201 10.1182/blood.v99.10.3505

[14] N. Win , E. Lee , M. Needs , L.‐W. Chia , and R. Stasi , “Measurement of Macrophage Marker in Hyperhaemolytic Transfusion Reaction: A Case Report,” Transfusion Medicine 22, no. 2 (2012): 137–141. 22233101 10.1111/j.1365-3148.2011.01131.x

[15] B. Cantinieaux , A. Janssens , J. R. Boelaert , et al., “Ferritin‐Associated Iron Induces Neutrophil Dysfunction in Hemosiderosis,” Journal of Laboratory and Clinical Medicine 133, no. 4 (1999): 353–361. 10218766 10.1016/s0022-2143(99)90066-5

[16] T. Miura and T. Ogiso , “Lipid Peroxidation of the Erythrocyte Membrane Caused by Stimulated Polymorphonuclear Leukocytes in the Presence of Ferritin,” Chemical and Pharmaceutical Bulletin 39, no. 6 (1991): 1507–1509. 1934170 10.1248/cpb.39.1507

[17] P. Biemond , A. J. Swaak , H. G. van Eijk , and J. F. Koster , “Superoxide Dependent Iron Release From Ferritin in Inflammatory Diseases,” Free Radical Biology & Medicine 4, no. 3 (1988): 185–198. 2833431 10.1016/0891-5849(88)90026-3

[18] J. W. Weisel , “Fibrinogen and Fibrin,” in Fibrous Proteins: Coiled‐Coils, Collagen and Elastomers [Advances in Protein Chemistry] (Elsevier, 2005), 247–299. 10.1016/S0065-3233(05)70008-5 15837518

[19] M. W. Mosesson , “Fibrinogen and Fibrin Structure and Functions,” Journal of Thrombosis and Haemostasis 3, no. 8 (2005): 1894–1904. 16102057 10.1111/j.1538-7836.2005.01365.x

[20] T. P. Ugarova and V. P. Yakubenko , “Recognition of Fibrinogen by Leukocyte Integrins,” Annals of the New York Academy of Sciences 936 (2001): 368–385. 11460493 10.1111/j.1749-6632.2001.tb03523.x

[21] S. T. Fan and T. S. Edgington , “Integrin Regulation of Leukocyte Inflammatory Functions. CD11b/CD18 Enhancement of the Tumor Necrosis Factor‐Alpha Responses of Monocytes,” Journal of Immunology 150, no. 7 (1993): 2972–2980. 8095957

[22] R. L. Perez , J. D. Ritzenthaler , and J. Roman , “Transcriptional Regulation of the Interleukin‐1beta Promoter via Fibrinogen Engagement of the CD18 Integrin Receptor,” American Journal of Respiratory Cell and Molecular Biology 20, no. 5 (1999): 1059–1066. 10226077 10.1165/ajrcmb.20.5.3281

[23] W. F. Skogen , R. M. Senior , G. L. Griffin , and G. D. Wilner , “Fibrinogen‐Derived Peptide B Beta 1‐42 Is a Multidomained Neutrophil Chemoattractant,” Blood 71, no. 5 (1988): 1475–1479. 3359049

[24] R. J. Fish and M. Neerman‐Arbez , “Fibrinogen Gene Regulation,” Thrombosis and Haemostasis 108, no. 3 (2012): 419–426. 22836683 10.1160/TH12-04-0273

[25] G. D. O. Lowe , “Circulating Inflammatory Markers and Risks of Cardiovascular and Non‐Cardiovascular Disease,” Journal of Thrombosis and Haemostasis 3, no. 8 (2005): 1618–1627. 16102027 10.1111/j.1538-7836.2005.01416.x

[26] R. F. Kraus , M. A. Gruber , and M. Kieninger , “The Influence of Extracellular Tissue on Neutrophil Function and Its Possible Linkage to Inflammatory Diseases,” Immunity, Inflammation and Disease 9, no. 4 (2021): 1237–1251. 34115923 10.1002/iid3.472 PMC8589351

[27] N. Doblinger , A. Bredthauer , M. Mohrez , et al., “Impact of Hydroxyethyl Starch and Modified Fluid Gelatin on Granulocyte Phenotype and Function,” Transfusion 59, no. 6 (2019): 2121–2130. 30934131 10.1111/trf.15279

[28] D. Pai , M. Gruber , S.‐M. Pfaehler , A. Bredthauer , K. Lehle , and B. Trabold , “Polymorphonuclear Cell Chemotaxis and Suicidal NETosis: Simultaneous Observation Using fMLP, PMA, H7, and Live Cell Imaging,” Journal of Immunology Research 2020 (2020): 1415947. 32879894 10.1155/2020/1415947 PMC7448108

[29] Ibidi GmbH , μ‐Slide Chemotaxis [Abrufdatum: 02.05.2022] (Verfügbar unter, 2022), https://ibidi.com/channel‐slides/9‐slide‐chemotaxis‐ibitreat.html.

[30] M. R. Ling , I. L. C. Chapple , A. J. Creese , and J. B. Matthews , “Effects of C‐Reactive Protein on the Neutrophil Respiratory Burst In Vitro,” Innate Immunity 20, no. 4 (2014): 339–349. 23839528 10.1177/1753425913493199

[31] C. Zouki , B. Haas , J. S. Chan , L. A. Potempa , and J. G. Filep , “Loss of Pentameric Symmetry of C‐Reactive Protein Is Associated With Promotion of Neutrophil‐Endothelial Cell Adhesion,” Journal of Immunology (Baltimore, Md: 1950) 167, no. 9 (2001): 5355–5361. 11673552 10.4049/jimmunol.167.9.5355

[32] B. Vulesevic , S. S. Lavoie , P.‐E. Neagoe , et al., “CRP Induces NETosis in Heart Failure Patients With or Without Diabetes,” Immunohorizons 3, no. 8 (2019): 378–388. 31399487 10.4049/immunohorizons.1900026

[33] V. Brinkmann , U. Reichard , C. Goosmann , et al., “Neutrophil Extracellular Traps Kill Bacteria,” Science 303, no. 5663 (2004): 1532–1535. 15001782 10.1126/science.1092385

[34] J. W. Yoon , M. V. Pahl , and N. D. Vaziri , “Spontaneous Leukocyte Activation and Oxygen‐Free Radical Generation in End‐Stage Renal Disease,” Kidney International 71, no. 2 (2007): 167–172. 17136029 10.1038/sj.ki.5002019

[35] S. W. Smail , E. Babaei , and K. Amin , “Hematological, Inflammatory, Coagulation, and Oxidative/Antioxidant Biomarkers as Predictors for Severity and Mortality in COVID‐19: A Prospective Cohort‐Study,” International Journal of General Medicine 16 (2023): 565–580. 36824986 10.2147/IJGM.S402206 PMC9942608

[36] M. Locke , R. J. Francis , E. Tsaousi , and C. Longstaff , “Fibrinogen Protects Neutrophils From the Cytotoxic Effects of Histones and Delays Neutrophil Extracellular Trap Formation Induced by Ionomycin,” Scientific Reports 10, no. 1 (2020): 11694. 32678135 10.1038/s41598-020-68584-0 PMC7366688

[37] C. Y. Han , T. J. Pichon , X. Wang , et al., “Leukocyte Activation Primes Fibrinogen for Proteolysis by Mitochondrial Oxidative Stress,” Redox Biology 51 (2022): 102263. 35158163 10.1016/j.redox.2022.102263 PMC8844908

[38] C. Rubel , G. C. Fernández , G. Dran , M. B. Bompadre , M. A. Isturiz , and M. S. Palermo , “Fibrinogen Promotes Neutrophil Activation and Delays Apoptosis,” Journal of Immunology 166, no. 3 (2001): 2002–2010. 10.4049/jimmunol.166.3.2002 11160249

[39] R. F. Kraus and M. A. Gruber , “Neutrophils‐From Bone Marrow to First‐Line Defense of the Innate Immune System,” Frontiers in Immunology 12 (2021): 767175. 35003081 10.3389/fimmu.2021.767175 PMC8732951

[40] F. S. Lakschevitz , S. Hassanpour , A. Rubin , N. Fine , C. Sun , and M. Glogauer , “Identification of Neutrophil Surface Marker Changes in Health and Inflammation Using High‐Throughput Screening Flow Cytometry,” Experimental Cell Research 342, no. 2 (2016): 200–209. 26970376 10.1016/j.yexcr.2016.03.007

[41] I. K. Quaye , “Haptoglobin, Inflammation and Disease,” Transactions of the Royal Society of Tropical Medicine and Hygiene 102, no. 8 (2008): 735–742. 18486167 10.1016/j.trstmh.2008.04.010

[42] S. Janciauskiene , S. Wrenger , S. Immenschuh , et al., “The Multifaceted Effects of Alpha1‐Antitrypsin on Neutrophil Functions,” Frontiers in Pharmacology 9 (2018): 341. 29719508 10.3389/fphar.2018.00341 PMC5914301

[43] R. Badolato , J. M. Wang , W. J. Murphy , et al., “Serum Amyloid A Is a Chemoattractant: Induction of Migration, Adhesion, and Tissue Infiltration of Monocytes and Polymorphonuclear Leukocytes,” Journal of Experimental Medicine 180, no. 1 (1994): 203–209. 7516407 10.1084/jem.180.1.203 PMC2191543

[44] R. Badolato , J. M. Wang , S. L. Stornello , A. N. Ponzi , M. Duse , and T. Musso , “Serum Amyloid A Is an Activator of PMN Antimicrobial Functions: Induction of Degranulation, Phagocytosis, and Enhancement of Anti‐Candida Activity,” Journal of Leukocyte Biology 67, no. 3 (2000): 381–386. 10733099 10.1002/jlb.67.3.381

[45] T. S. Liang , J. M. Wang , P. M. Murphy , and J. L. Gao , “Serum Amyloid A Is a Chemotactic Agonist at FPR2, a Low‐Affinity N‐Formylpeptide Receptor on Mouse Neutrophils,” Biochemical and Biophysical Research Communications 270, no. 2 (2000): 331–335. 10753626 10.1006/bbrc.2000.2416

[46] S. L. Silverman , E. S. Cathcart , M. Skinner , and A. S. Cohen , “The Degradation of Serum Amyloid A Protein by Activated Polymorphonuclear Leucocytes: Participation of Granulocytic Elastase,” Immunology 46, no. 4 (1982): 737–744. 6921153 PMC1555477

[47] M. Weckmann , T. Becker , G. Nissen , M. Pech , and M. V. Kopp , “SiMA: A Simplified Migration Assay for Analyzing Neutrophil Migration,” Cytometry. Part A 91, no. 7 (2017): 675–685. 10.1002/cyto.a.23114 28544679

[48] R. Grieshaber‐Bouyer , F. A. Radtke , P. Cunin , et al., “The Neutrotime Transcriptional Signature Defines a Single Continuum of Neutrophils Across Biological Compartments,” Nature Communications 12, no. 1 (2021): 2856. 10.1038/s41467-021-22973-9 PMC8129206 34001893

[49] E. Kolaczkowska and P. Kubes , “Neutrophil Recruitment and Function in Health and Inflammation,” Nature Reviews. Immunology 13, no. 3 (2013): 159–175. 10.1038/nri3399 23435331

[50] D. Fröhlich , S. Wittmann , G. Rothe , G. Schmitz , and K. Taeger , “Thiopental Impairs Neutrophil Oxidative Response by Inhibition of Intracellular Signalling,” European Journal of Anaesthesiology 19, no. 7 (2002): 474–482. 12113609 10.1017/s0265021502000789

[51] J. Rimboeck , M. Gruber , and S. Wittmann , “Is the In Vitro Observed NETosis the Favored Physiological Death of Neutrophils or Mainly Induced by an Isolation Bias?,” International Journal of Molecular Sciences 24, no. 8 (2023): 7368. 37108529 10.3390/ijms24087368 PMC10138317

[52] T. Hundhammer , M. Gruber , and S. Wittmann , “Paralytic Impact of Centrifugation on Human Neutrophils,” Biomedicine 10, no. 11 (2022): 2896. 10.3390/biomedicines10112896 PMC9687505 36428463

[53] G. Kamoshida , T. Kikuchi‐Ueda , S. Nishida , et al., “Spontaneous Formation of Neutrophil Extracellular Traps in Serum‐Free Culture Conditions,” FEBS Open Bio 7, no. 6 (2017): 877–886. 10.1002/2211-5463.12222 PMC5458474 28593142

[54] R. Alipour , A. Fatemi , F. Alsahebfosul , A. Andalib , and A. Pourazar , “Autologous Plasma Versus Fetal Calf Serum as a Supplement for the Culture of Neutrophils,” BMC Research Notes 13, no. 1 (2020): 39. 31969182 10.1186/s13104-020-4902-z PMC6977324

[55] H. Parker , M. Dragunow , M. B. Hampton , A. J. Kettle , and C. C. Winterbourn , “Requirements for NADPH Oxidase and Myeloperoxidase in Neutrophil Extracellular Trap Formation Differ Depending on the Stimulus,” Journal of Leukocyte Biology 92, no. 4 (2012): 841–849. 22802447 10.1189/jlb.1211601

[56] T. Hoppenbrouwers , A. S. A. Autar , A. R. Sultan , et al., “In Vitro Induction of NETosis: Comprehensive Live Imaging Comparison and Systematic Review,” PLoS One 12, no. 5 (2017): e0176472. 28486563 10.1371/journal.pone.0176472 PMC5423591

