# Supplementary material for: Influence of Acute Phase Proteins on Neutrophil Function In Vitro
Source: FASEB Bioadv. 2025 Oct 19;7(10):e70062. doi: 10.1096/fba.2025-00148 (PMC12535726; doi:10.1096/fba.2025-00148)
Supplement: Supplementary file 1 — Table S1: Demographic data of the healthy PMN donors for the live cell imaging. [file FBA2-7-e70062-s001.pdf]

Table S1: Demographic data of the healthy PMN donors for the live cell imaging

Parameter	Age [a]	sex [male/female)	Height [cm]	Weight [kg]	Smoker	Daily intake of medical drugs	Medical drugs	Allergies	Allergy
	23	male	185	78	no	yes	Ibuprofene	yes	Allergic rhinitis
	22	female	168	60	no	yes	Salmeterol/Fluticason	yes	Allergic rhinitis
	28	male	178	81	no	no		no	
	23	female	182	70	no	no		no	
	31	male	177	71	no	no		no	
	22	female	175	58	no	no		no	
	24	male	182	78	no	yes	Oral contraceptivum	no	
	21	male	182	70	no	no		yes	Allergic rhinitis
	53	male	183	91	no	no		no	
	20	female	169	50	no	no		no	
	56	male	175	76	no	yes	Pantoprazole	no	
Mean	29,4		177,8	71,2					

0	0
0	0
0	0
0	0
